# Hyperlipidemia is negatively associated with pregnancy outcomes in patients following frozen‐thawed embryo transfer: A retrospective study

**DOI:** 10.1002/hsr2.1947

**Published:** 2024-03-04

**Authors:** Fang Yang, Wei Mao, Yan‐Mei Ge, Xia Zhao, Jie Song, Jin‐Chun Lu, Yuan‐Jiao Liang

**Affiliations:** ^1^ Center for Reproductive Medicine, Zhongda Hospital Southeast University Nanjing Jiangsu China; ^2^ Wuxi Maternal and Child Health Hospital, Wuxi School of Medicine Jiangnan University Wuxi Jiangsu China

**Keywords:** endometrial preparation protocol, frozen‐thawed embryo transfer, hyperlipidemia, pregnancy outcome

## Abstract

**Background and Aims:**

It is demonstrated that lipid metabolism disorders are associated with the reproductive performances of assisted reproductive technology. However, it is little known whether hyperlipidemia is associated with the endometrial receptivity and pregnancy outcomes of patients undergoing frozen‐thawed embryo transfer (FET).

**Methods:**

This was a retrospective analysis involving 554 infertile women undergoing FET. The patients were divided into the hyperlipidemia group (*n* = 224) and control group (*n* = 320) based on the levels of serum lipids. The clinical and laboratory indexes between the two groups were compared. Meanwhile, the stratified analysis based on body mass index (BMI) and endometrial preparation protocols was performed. The independent samples *t*‐test, Mann−Whitney *U* test, *χ*2 test and multiple logistic regression analysis were used to compare and analyze the data.

**Results:**

The patients with hyperlipidemia had significantly higher serum lipids levels and BMI and lower clinical pregnancy and implantation rates than those with normal blood lipids (*p* < 0.05). The impact of hyperlipidemia on pregnancy outcomes was independent of BMI. The multiple logistic regression analysis showed that higher cholesterol was associated with lower pregnancy rate and implantation rate (*p* < 0.05). Regardless of blood lipid levels, the patients undergoing the hormone replacement therapy (HRT) protocol had higher estradiol levels and lower progesterone levels compared with the stimulated cycles (STC) (*p* < 0.05). Moreover, the clinical pregnancy rate and implantation rate of the HRT protocol were higher than those of the STC, although there was no significant difference between the two.

**Conclusion:**

Hyperlipidemia especially higher cholesterol has a negative effect on the pregnancy outcomes of the patients undergoing FET. Actively implementing lipid‐lowering treatment and the HRT protocol seem more friendly for these patients.

## 1 INTRODUCTION

An unhealthy life‐style has recently increased the incidence of hyperlipidemia, and the prevalence of hyperlipidemia in adults increased from 18.6% in 2002 to 40.4% in 2012 in China.^1^ More and more evidence has demonstrated that lipid metabolism disorders are associated with the reproductive performances of assisted reproductive technology (ART).

A study aimed at infertility patients receiving donor eggs found that dyslipidemia significantly reduced their biochemical pregnancy rate, embryo implantation rate, and live birth rate.^2^ A retrospective real‐world analysis involving 5030 infertile women found that the women with increased serum lipid concentrations had a significantly higher late miscarriage rate, lower term birth rate, and lower live birth rate.^3^ A study including 468 patients with polycystic ovarian syndrome (PCOS) found that dyslipidemia, especially the increase of serum triglyceride (TG) level, had a negative impact on the implantation rate, biochemical pregnancy rate, clinical pregnancy rate, and live birth rate.^4^ It was also reported that serum total cholesterol (TC) levels were significantly negatively associated with the cumulative live birth rate (cLBR).^5^ A study of 1132 patients without PCOS proved that dyslipidemia was negatively associated with the cLBR. However, there was no significant difference in the live birth rate of fresh cycles between dyslipidemia and control groups.^6^ A significant regression study focused on dyslipidemia patients with or without PCOS found that the cumulative pregnancy outcomes of 1470 patients with PCOS were similar in dyslipidemia and control groups. Similarly, there was no significant difference in the cumulative pregnancy outcomes of 3232 patients without PCOS between dyslipidemia and control groups.^5^ Sun et al.^7^ also reported no difference in pregnancy and miscarriage rates between women with and without hyperlipidemia. The inconsistent results of the above studies may be related to the fact that most studies focus on the fresh cycle of ART, which includes many confounding factors such as the higher dosage of gonadotropin (Gn), different ovarian stimulation protocols, higher estrogen levels on the human chorionic Gn (HCG) trigger day, and so forth.

There have been few studies investigating the relationship between dyslipidemia and endometrial receptivity in the frozen‐thawed embryo transfer (FET) cycle. Compared to the fresh cycle, the FET cycle may be a better approach for studying the potential involvement of the endometrium, as the obtained results are free from the influences of exogenous hormones. This study retrospectively analyzed the data from FET cycles to reveal whether hyperlipidemia was related to endometrial receptivity. To avoid the impact of embryonic factors on the results, only cleavage embryos with good quality were transplanted. In addition, this study compared the effects of dyslipidemia on laboratory indexes and pregnancy outcomes in women undergoing FET and performed stratified analysis based on body mass index (BMI) and endometrial preparation protocols, as BMI could reflect the degree of obesity, and different endometrial preparation protocols were related to the source of steroid hormones. It was reported that both high‐density lipoprotein (HDL) and low‐density lipoprotein (LDL) could transport cholesterol to ovarian tissue, and that cholesterol was a substrate of steroidogenesis.^8^ The hormone replacement therapy (HRT) protocol directly provided estradiol, while the estradiol in the stimulated cycle (STC) was converted from cholesterol. The implementation of this study may provide appropriate intervention measures for women undergoing the FET cycle.

## 2 MATERIALS AND METHODS

### 2.1 Study population

The clinical data of 544 women undergoing FET treatment in our hospital between August 2017 and October 2022 were analyzed retrospectively. The implementation of ART was approved by the Reproductive Medicine Ethics Committee of Zhongda Hospital affiliated to Southeast University (Reproduction No. 2015‐1), and all patients signed the informed consent. The inclusion criteria of the patients included: (1) females aged 20−38 years old, (2) normal ovarian reserve. Exclusion criteria included: (1) severe adenomyosis or endometriosis, (2) intrauterine adhesions, (3) chronic endometritis, (4) submucous myomas or big myomas that distort the uterine cavity, (5) severe hydrosalpinx, (6) a history of recurrent pregnancy loss, (7) chromosome abnormality or other genetic mutations, (8) hypertension, diabetes or other conditions that may disturb the outcomes of ART.

All the patients were divided into dyslipidemia (hyperlipidemia) and normal (control) groups. The diagnostic criteria of hyperlipidemia were based on the 2016 guidelines for the prevention and treatment of hyperlipidemia in Chinese adults.^1^ Hyperlipidemia was defined as having at least one of the following indexes: TC ≥ 5.18 mmol/L or ≥200 mg/dL, LDL cholesterol (LDL‐C) ≥ 3.37 mmol/L or ≥130 mg/dL, HDL cholesterol (HDL‐C) < 1.04 mmol/L or <40 mg/dL, and TG ≥ 1.7 mmol/L or ≥150 mg/dL.

### 2.2 Endometrial preparation

In HRT cycles, 4 mg of oral estradiol valerate (Progynova; Bayer, Germany) was administered per day from Day 2–4 of the menstrual cycle. The patients were evaluated by transvaginal ultrasound 7 days later to adjust the dosage of estradiol based on the endometrial thickness. A supplementation of vaginal estradiol was added if the endometrial thickness continued to be unsatisfactory. When the endometrial thickness reached 8 mm, and serum progesterone level was below 1.5 ng/mL, intramuscular administration of 40 mg progesterone (Zhejiang Xianju Pharmaceutical Co., Ltd) or vaginal supplementation with 90 mg progesterone (8% Crinone, Merck‐Serono) was added before transferring cleavage stage embryos.

In STCs, the patients received letrozole (Jiangsu Hengrui Pharmaceutical Co., Ltd) 2.5 mg per day from Day 2–4 of the menstrual cycle, and human menopausal Gn (hMG; Lebaode, Livzon Pharmaceutical Group Inc.) was given according to the speed of follicular growth which was evaluated by transvaginal ultrasound. The dosage of Gn was adjusted according to the BMI (weight [kg]/height [m]^2^) and ovarian reserve of the patients. When the diameter of follicles was ≥18 mm, 10,000 IU of human chorionic Gn (HCG; Livzon Pharmaceutical Group Inc.) were injected to induce oocyte ovulation. FET was carried out 3 days after oocyte ovulation.

To reduce divergence, only cleavage embryos were selected for FET in this retrospective study. One to two embryos with good quality were transferred into the uterus, and then the patients were given routine corpus luteum support. The serum HCG levels of the patients were detected 14 days after embryo transfer. If the serum HCG level was higher than 50 U/L, the patient was regarded as a biochemical pregnancy. Clinical pregnancy was confirmed by ultrasound, and one or two gestational sacs were visible approximately 4 weeks after embryo transfer.

### 2.3 Laboratory and clinical indexes

Blood samples for the detection of lipids such as TC, LDL‐C, HDL‐C, and TG and basal reproductive hormones such as anti‐Müllerian hormone (AMH), follicle‐stimulating hormone (FSH), luteinizing hormone (LH), estradiol (E2) and progesterone (P) were obtained from Day 2−4 of the menstrual cycle. Serum E2 and P levels were also detected on the hCG trigger day of the STC or the progesterone administration day of the HRT cycle. Commercially available kits for the determinations of serum FSH, LH, E2, P, and AMH were purchased from Abbott Laboratories, Inc. USA, and serum FSH, LH, E2, P, and AMH levels were determined by chemiluminescence assay using an automated Abbott Architect i1000 system (Abbott Laboratories, Inc.). Commercially available kits for the determinations of TG, TC, LDL‐C, and HDL‐C were purchased from Shanghai Zhicheng Biotechnology Co. Calibration and quality control products were purchased from Randox Laboratories Ltd. The determinations of serum lipids were carried out using a Beckman Coulter AU5800 automatic biochemistry analyzer (Beckman Coulter, Inc.). The intra‐laboratory quality control of all testing indexes was carried out with each batch of samples, and inter‐laboratory external quality assessment was participated in twice a year. All testing indexes were under control. The indexes of clinical outcomes such as pregnancy rate (the number of cycles achieving clinical pregnancy/the number of embryo transfer cycles × 100%), implantation rate (the number of implanted embryos/the number of embryos transferred × 100%), and miscarriage rate (the number of miscarriage cycles/the number of clinical pregnancy cycles × 100%) were calculated.

### 2.4 Statistical analysis

Statistical analysis was performed with SPSS 22.0 (SPSS Inc.). The measurement data were first performed by one‐sample nonparametric tests (Kolmogorov−Smirnov test) to determine whether they were normal distribution. The data conforming to normal distribution were expressed as mean ± standard deviation (x ± s), and those conforming to non‐normal distribution were expressed as median [P_25_, P_75_]. The comparisons between control and hyperlipidemia groups were analyzed by independent samples *t*‐test for normal distribution data or Mann−Whitney *U* test for non‐normal distribution data. The count data were presented as percentages, and the comparisons between the control and hyperlipidemia groups were analyzed by the *χ* ^2^ test. The multiple logistic regression analysis was used to analyze the impacts of blood lipids on the clinical outcomes of patients undergoing FET. They all performed the 2‐sided test. *p* ≤ 0.05 was considered to be statistically significant.

## 3 RESULTS

### 3.1 Comparisons of basic clinical information between control and hyperlipidemia groups in the patients undergoing FET

The comparisons of basic clinical information between control and hyperlipidemia groups are shown in Table 1. The patients with hyperlipidemia had significantly abnormal serum lipids and greater BMI compared to those with normal blood lipids (*p* < 0.05). There were no significant differences in age, basal FSH, basal LH, basal E2, basal progesterone, AMH, endometrial preparation protocols and duration from lipids detection to FET between the two groups (*p* > 0.05).

**Table 1:** Comparisons of basic clinical information between control and hyperlipidemia groups in the patients undergoing FET.

Variables	Control group (*n* = 320)	Hyperlipidemia group (*n* = 224)	*p*
Age (years old)	30.3 ± 3.4	30.7 ± 3.5	0.16
BMI (kg/m^2^)	22.3 ± 3.4	23.8 ± 3.9	<0.001
BMI distribution, *n* (%)			<0.001
BMI < 24 kg/m^2^	232/320 (72.5)	120/224 (53.6)	
BMI ≥ 24 kg/m^2^	88/320 (27.5)	104/224 (46.4)	
AMH (ng/mL)	5.0 ± 3.6	5.1 ± 3.2	0.75
Basal FSH (mIU/mL)	6.2 ± 1.3	6.3 ± 1.3	0.58
Basal LH (mIU/mL)	6.9 ± 2.0	6.9 ± 2.0	0.79
Basal E2 (pg/mL)	40.5 ± 12.4	40.1 ± 14.0	0.73
Basal P (ng/mL)	0.46 ± 0.28	0.43 ± 0.27	0.24
TC (mmol/L)	4.20 ± 0.63	5.21 ± 0.85	<0.001
TG (mmol/L)	0.98 [0.68−1.24]	1.74 [1.11−2.11]	<0.001
HDL (mmol/L)	1.57 ± 0.42	1.47 ± 0.44	0.006
LDL (mmol/L)	2.34 ± 0.49	3.01 ± 0.72	<0.001
Endometrial preparation protocol, *n* (%)			0.80
HRT	212/320 (66.3)	146/224 (65.2)	
STC	108/320 (33.7)	78/224 (34.8)	
Duration from lipids detection to FET (days)	15.9 ± 2.4	16.1 ± 3.1	0.35

*Note*: The measurement data were first performed by one‐sample nonparametric tests (Kolmogorov−Smirnov test) to determine whether they were normal distribution. The data conforming to normal distribution were expressed as mean ± standard deviation (x ± s), and those conforming to non‐normal distribution were expressed as median [P25, P75]. The comparisons between control and hyperlipidemia groups were analyzed by independent samples *t*‐test for normal distribution data or Mann−Whitney *U* test for non‐normal distribution data. The count data were presented as percentages, and the comparisons between the control and hyperlipidemia groups were analyzed by the *χ* ^2^ test. A *p* ≤ 0.05 was considered statistically significant.

Abbreviations: AMH, anti‐Müllerian hormone; BMI, body mass index; E2, estradiol; FET, frozen‐thawed embryo transfer; FSH, follicle stimulating hormone; HDL, high‐density lipoprotein cholesterol; HRT, hormone replacement therapy; LDL, low‐density lipoprotein cholesterol; LH, luteinizing hormone; P, progesterone; TC, total cholesterol; TG, triglyceride; STC, stimulated cycles.

### 3.2 Comparisons of clinical outcomes between hyperlipidemia and control groups in the patients undergoing FET

The clinical outcomes of the patients undergoing FET are shown in Table 2. There were no significant differences in endometrial thickness, estradiol and progesterone levels on the progesterone administration or HCG trigger day and miscarriage rate between hyperlipidemia and control groups (*p* > 0.05). However, the patients with hyperlipidemia had significantly lower clinical pregnancy and implantation rates than those with normal blood lipids (*p* < 0.05).

**Table 2:** Comparisons of clinical outcomes between hyperlipidemia and control groups in the patients undergoing FET.

Variable	Control group (*n* = 320)	Hyperlipidemia group (*n* = 224)	*p*
Endometrial thickness (mm)	10.4 ± 1.9	10.3 ± 2.0	0.61
E2 levels on the progesterone administration or HCG trigger day (pg/mL)	394.4 ± 199.6	379.4 ± 220.0	0.41
P levels on the progesterone administration or HCG trigger day (pg/mL)	0.51 ± 0.4	0.48 ± 0.35	0.34
Clinical pregnancy rate, *n* (%)	193/320 (60.3)	115/224 (51.3)	0.04
Implantation rate, *n* (%)	236/513 (46.0)	133/357 (37.2)	<0.001
Miscarriage rate, *n* (%)	20/193 (10.4)	13/115 (11.3)	0.80

*Note*: The measurement data conformed to the normal distribution and were presented as mean ± standard deviation (x ± s). The comparisons between control and hyperlipidemia groups were analyzed by independent samples *t*‐test. The count data were presented as percentages, and the comparisons between the control and hyperlipidemia groups were analyzed by the *χ* ^2^ test. A *p* ≤ 0.05 was considered statistically significant.

Abbreviations: E2, estradiol; FET, frozen‐thawed embryo transfer; HCG, human chorionic gonadotropin; P, progesterone.

### 3.3 Stratified comparisons of clinical indexes in patients with different blood lipids levels based on BMI

To investigate whether the impact of hyperlipidemia on reproductive outcomes was related to obesity, we further compared clinical indexes based on BMI stratification. A BMI less than 24 kg/m^2^ was considered normal, while a BMI ≥ 24 kg/m^2^ was considered overweight or obese. As shown in Table 3, regardless of BMI, serum E2 levels on the progesterone administration or HCG trigger day, the clinical pregnancy rate and implantation rate of women with hyperlipidemia were lower than those of women with normal blood lipids. However, only the implantation rate in the patients with BMI < 24 kg/m^2^ showed a significant difference between the two groups (*p* < 0.05). There were no significant differences in age, AMH, endometrial thickness, serum P levels on the progesterone administration or HCG trigger day, and miscarriage rate between groups (*p* > 0.05).

**Table 3:** Comparisons of clinical indexes in patients with different blood lipids levels based on BMI.

Variables	BMI < 24 kg/m^2^ (*n* = 352)		BMI ≥ 24 kg/m^2^ (*n* = 192)	
	Control group (*n* = 232)	Hyperlipidemia group (*n* = 120)	Control group (*n* = 88)	Hyperlipidemia group (*n* = 104)
Age (years old)	30.0 ± 3.3	30.4 ± 3.9	31.2 ± 3.3	31.1 ± 3.0
AMH (ng/mL)	4.9 ± 3.5	5.3 ± 3.1	5.2 ± 3.8	4.8 ± 3.3
Endometrium thickness (mm)	10.3 ± 2.0	10.0 ± 1.9	10.6 ± 1.8	10.8 ± 2.1
E2 levels on the progesterone administration or HCG trigger day (pg/mL)	384.5 ± 186.7	373.0 ± 211.8	420.5 ± 229.3	386.8 ± 230.0
P levels on the progesterone administration or HCG trigger day (ng/mL)	0.54 ± 0.42	0.53 ± 0.39	0.43 ± 0.34	0.41 ± 0.30
Clinical pregnancy rate, *n* (%)	143/232 (61.6)	62/120 (51.7)	50/88 (56.8)	53/104 (51.0)
Implantation rate, *n* (%)	176/378 (46.6)	70/196 (35.7)*	60/135 (44.4)	63/161 (39.1)
Miscarriage rate, *n* (%)	15/143 (10.5)	7/62 (11.3)	5/50 (10)	6/53 (11.3)

*Note*: The measurement data conformed to the normal distribution and were presented as mean ± standard deviation (x ± s). The comparisons between control and hyperlipidemia groups were analyzed by independent samples *t*‐test. The count data were presented as percentages, and the comparisons between the control and hyperlipidemia groups were analyzed by the *χ* ^2^ test.

* Compared with the control in the same group, *p* < 0.05.

### 3.4 Multiple logistic regression analysis between blood lipids and the clinical outcomes of patients undergoing FET

Multiple logistic regression analysis showed that serum TC was significantly associated with lower pregnancy and implantation rates (*p* < 0.05, Table 4). Serum TG, HDL, and LDL were not associated with the clinical outcomes of patients undergoing FET.

**Table 4:** Multiple logistic regression analysis between blood lipids and the clinical outcomes of patients undergoing FET (OR [95% CI], *p* Value).

Variables	Pregnancy rate	Implantation rate	Miscarriage rate
TG	0.837 (0.673‐1.041) *p* = 0.11	0.929 (0.788−1.096) *p* = 0.38	0.753 (0.437−1.298) *p* = 0.31
TC	0.694 (0.519‐0.930) *p* = 0.014	0.700 (0.555−0.882) *p* = 0.002	1.071 (0.579−1.980) *p* = 0.83
HDL	1.135 (0.706‐1.825) *p* = 0.60	1.379 (0.949−2.005) *p* = 0.09	0.679 (0.263−1.757) *p* = 0.43
LDL	0.889 (0.623‐1.270) *p* = 0.52	0.973 (0.737−1.284) *p* = 0.84	1.249 (0.567−2.751) *p* = 0.58

*Note*: The multiple logistic regression analysis was conducted using pregnancy rate, implantation rate, or miscarriage rate as dependent variables and TG, TC, HDL, and LDL as covariates. The results were expressed in odd ratio (OR) and 95% confidence interval (CI). A *p* ≤ 0.05 was considered statistically significant.

Abbreviations: HDL, high‐density lipoprotein cholesterol; LDL, low‐density lipoprotein cholesterol; TC, total cholesterol; TG, triglyceride.

### 3.5 Stratified comparisons of clinical indexes in patients with different endometrial preparation protocols based on blood lipid levels

The comparisons of clinical indexes in patients with different endometrial preparation protocols based on blood lipid levels are shown in Table 5. There were no significant differences in age, AMH, and endometrial thickness between groups (*p* > 0.05). Regardless of blood lipid levels, the HRT group had higher estradiol levels and lower progesterone levels than the STC group (*p* < 0.05). Although the clinical pregnancy rate and implantation rate of the HRT group were higher than those of the STC group in patients with different blood lipid levels, there was no significant difference between the two groups. In addition, we compared the clinical indexes of women with different blood lipid levels under the same endometrial preparation protocol and found that serum E2 levels on the progesterone administration or HCG trigger day and the implantation rate of hyperlipidemic women undergoing the STC were significantly lower than in women with normal blood lipids (*p* < 0.05).

**Table 5:** Stratified comparisons of clinical indexes in patients with different endometrial preparation protocols based on blood lipids levels.

Variables	Control group (*n* = 320)		Hyperlipidemia group (*n* = 224)	
	HRT (*n* = 212)	STC (*n* = 108)	HRT (*n* = 146)	STC (*n* = 78)
Age (years old)	30.1 ± 3.6	30.7 ± 2.9	30.7 ± 3.5	30.9 ± 3.5
AMH (ng/mL)	5.2 ± 3.8	4.6 ± 3.0	5.3 ± 3.3	4.6 ± 2.8
Endometrium thickness (mm)	10.5 ± 1.8	10.3 ± 2.2	10.3 ± 2.2	10.4 ± 1.8
E2 levels on the progesterone administration or HCG trigger day (pg/mL)	412.0 ± 219.1	359.9 ± 149.6*	415.6 ± 252.5	311.6 ± 114.1*,**
P levels on the progesterone administration or HCG trigger day (ng/mL)	0.38 ± 0.34	0.76 ± 0.40*	0.34 ± 0.30	0.73 ± 0.31*
Clinical pregnancy rate, *n* (%)	130/212 (61.3)	63/108 (58.3)	78/146 (53.4)	37/78 (47.4)
Implantation rate, *n* (%)	158/337 (46.9)	78/176 (44.3)	90/236 (38.1)	43/121 (35.5)**
Miscarriage rate, *n* (%)	12/130 (9.2)	8/63 (12.7)	8/78 (10.3)	5/37 (13.5)

*Note*: The measurement data conformed to the normal distribution and were presented as mean ± standard deviation (x ± s). The comparisons between control and hyperlipidemia groups were analyzed by independent samples *t*‐test. The count data were presented as percentages, and the comparisons between the control and hyperlipidemia groups were analyzed by the *χ* ^2^ test.

Abbreviations: AMH, anti‐Müllerian hormone; E2, estradiol; HCG, human chorionic gonadotropin; HRT, hormone replacement therapy; P, progesterone; STC, stimulated cycles.

* Compared with the HRT in the same group, *p* < 0.05;

** Compared with the STC in the control group, *p* < 0.05.

## 4 DISCUSSION

The prevalence of hyperlipidemia in Chinese people has increased significantly, reaching 40.7% in 2016.^1^ Hyperlipidemia not only affects the cardiovascular system but also has adverse effects on the female reproductive system. Pugh et al.^9^ reported a study including 848 hyperlipidemia women and 380 control women and found a 19−36% decrease in the probability of conception per cycle for women with abnormal lipoprotein levels. It was reported that serum lipids levels were associated with embryo quality and pregnancy rates and that higher serum TG level was associated with a lower chance of live birth.^10^, ^11^ Some studies have confirmed that hyperlipidemia was negatively associated with the cLBR of patients undergoing in vitro fertilization or intracytoplasmic sperm injection.^5^, ^9^ It seemed that LDL had a negative impact on pregnancy outcomes, including lower pregnancy rate, lower live birth rate and higher miscarriage rate, while higher serum HDL levels were associated with better pregnancy outcomes.^12^

Embryo quality and endometrial receptivity are considered key factors for a successful pregnancy. Most studies have found an adverse effect of hyperlipidemia on embryos. However, the impact of hyperlipidemia on endometrial receptivity was rarely reported. Moreover, most studies on the relationship between dyslipidemia and assisted reproductive outcomes focus on the fresh cycle, which may be affected by many confounding factors, such as higher Gn dosage and estrogen levels. Therefore, this study mainly observed the effect of dyslipidemia on endometrial receptivity in the FET cycle. To avoid interference from embryo quality, good‐quality embryos were selected to transfer into the uterus in this study.

Our study found that there were no differences in age, AMH, basal FSH, LH, E2, and P levels, endometrium thickness, and E2 and P levels on the progesterone administration or HCG trigger day between hyperlipidemia and control groups. However, regardless of whether obesity or not, there were significant differences in pregnancy outcomes between the two groups, consistent with other studies.^9^, ^10^ Moreover, we found that serum TC was closely associated with lower pregnancy and implantation rates. Since all the patients received good embryos, it was speculated that hyperlipidemia might be a risk factor for endometrial receptivity. A large sample study involving 5030 infertile women found that the prevalence of type C endometrium was higher in women with hyperlipidemia, and their live birth rate was lower than that of the control group.^3^ Our study found similar results, and there was no significant difference in endometrial thickness between hyperlipidemia and control groups, indicating that hyperlipidemia might affect endometrial receptivity at the molecular level. Some studies indicated that blood cholesterol profiles fluctuated during the ovarian cycle to establish a receptive endometrium.^13^ However, how lipids affect endometrium is unclear.

Lipid molecules such as endocannabinoids, lysophosphatidic acid, and prostaglandins (PGs) have been reported to be of great value in endometrial receptivity. The PGs derived from fatty acids (FAs) are thought to be critical for the successful implantation of embryos. The concentrations of PGE2 and PGF2a in the endometrial fluid increased significantly during the window period of embryo implantation.^14^ PGF2a was able to enhance utero blood circulation by activating the mitogen‐activated protein kinase signaling pathway to stimulate the secretion of vascular endothelial growth factor.^15^ The patients with recurrent implantation failure exhibited aberrant synthesis of endometrial PGs such as PGE2 and PGF2a.^16^ It was reported that the rats fed a high‐cholesterol diet for 6 weeks had significantly higher serum TC, TG, and LDL levels and lower PGE2 levels than the control rats.^17^ Liu et al.^18^ verified that cholesterol could suppress flow‐mediated PGE2 release in the mice renal collecting duct via a p38‐dependent mechanism.

When we focused on endometrial preparation protocols, we found some interesting results. There was no significant difference in estrogen levels between the patients with and without hyperlipidemia in the HRT cycle, but the patients with hyperlipidemia had significantly lower estrogen levels in the STC, which was consistent with other studies.^19^, ^20^ Why do hyperlipidemic patients receiving the STC protocol have reduced serum estrogen levels? Some studies have reported this phenomenon, but its mechanism and impact are still unclear. As we all know, steroid hormones such as estrogen and progesterone are derived from lipids through the action of many enzymes and molecules. Theoretically, women with hyperlipidemia should have higher serum estrogen levels, but the results are the opposite. We speculate that the endogenous estrogen production pathway in women with hyperlipidemia may be abnormal, interfering with lipids' conversion to steroids. Previous studies found that PGs were closely related to steroids regulating molecules such as Cyp19 and Cyp11. It was verified that the expression of Cyp19 in human endometrial stromal cells (ESCs) was regulated by PGE2, and that PGE2‐stimulated Cyp19 and ERβ expression was mainly regulated by the p38 and JNK pathway.^21^ Jonczyk et al.^22^ observed a lower CYP11A1 expression after local PGF2a analogs injection in the middle‐stage corpus luteum of cows. These indicated that PGs could affect estrogen synthesis by regulating steroid‐related molecules and that the lower estrogen levels in hyperlipidemia patients' STC might contribute to abnormal PGs. It was reported that anandamide (AEA) inhibited the proliferation and differentiation of ESCs and that the inhibitory effect of AEA on cell decidualization might be mediated by its interaction with CYP19A3, resulting in interference with estradiol production signals.^23^ However, whether these molecules have abnormal expressions in the endometrium of dyslipidemia women needs to be further verified.

With the development of genetic testing technology, some genes related to endometrial receptivity have been well identified. A retrospective analysis of the endometrial receptivity array in women with reproductive dysfunction showed that 12 core genes were associated with lipid metabolism.^24^ A study aimed at gene expression datasets found that 40 upregulated and 21 downregulated transcripts were related to endometrial receptivity and some of them were regulated by sterol regulatory element binding protein 1, which was a key regulator of cholesterol homeostasis.^25^

In our study, the impacts of systemic diseases, uterine lesions, and genetic factors on the results were avoided by the strict inclusion criteria. Moreover, only cleavage embryos with good quality were transplanted to avoid the impact of embryonic factors. By taking these measures to minimize the impacts of confounding factors as much as possible, the conclusions drawn by our study should be relatively reliable. However, there are some limitations in our study. As a retrospective study, some confounding factors such as embryo quality, local endometrial blood flow, or immune factors may interfere with the results of our study. In addition, this study is only a phenomenon observation, and the specific mechanism by which hyperlipidemia affects pregnancy outcomes of the patients undergoing FET still needs to be further elucidated.

## 5 CONCLUSIONS

Our findings indicate that hyperlipidemia, especially higher cholesterol, has a negative effect on the pregnancy outcomes of the patients undergoing FET. The impact of hyperlipidemia on pregnancy outcomes may be related to endometrial preparation protocols. Compared with the HRT, the STC protocol can lead to a decrease in estrogen levels and an increase in progesterone levels, which may affect endometrial receptivity and have a negative effect on pregnancy outcomes. In addition to actively implementing lipid‐lowering treatment, the HRT protocol may be more suitable for hyperlipidemic patients undergoing FET, which requires more data support.

## AUTHOR CONTRIBUTIONS

**Fang Yang**: Conceptualization; formal analysis; writing—original draft. **Wei Mao**: Formal analysis. **Yan‐Mei Ge**: Data curation. **Xia Zhao**: Data curation. **Jie Song**: Data curation. **Jin‐Chun Lu**: Conceptualization; formal analysis; writing—review and editing. **Yuan‐Jiao Liang**: Resources; supervision.

## CONFLICT OF INTEREST STATEMENT

The authors declare no conflict of interest.

## TRANSPARENCY STATEMENT

The Jin‐Chun Lu and Yuan‐Jiao Liang affirm that this manuscript is an honest, accurate, and transparent account of the study being reported; that no important aspects of the study have been omitted; and that any discrepancies from the study as planned (and, if relevant, registered) have been explained.

## Data Availability

The data are available from the corresponding author on reasonable request.
